# Life-long oligodendrocyte development and plasticity

**DOI:** 10.1016/j.semcdb.2021.02.004

**Published:** 2021-08

**Authors:** Akiko Nishiyama, Takahiro Shimizu, Amin Sherafat, William D. Richardson

**Affiliations:** a Department of Physiology and Neurobiology, University of Connecticut, Storrs, CT 06269-3156, USA; b Wolfson Institute for Biomedical Research, University College London, Gower Street, London WC1E 6BT, UK

**Keywords:** NG2, myelin, PDGF, AMPA receptor, Adaptive myelination, Learning and memory

## Abstract

Oligodendrocyte precursor cells (OPCs) originate in localized germinal zones in the embryonic neural tube, then migrate and proliferate to populate the entire central nervous system, both white and gray matter. They divide and generate myelinating oligodendrocytes (OLs) throughout postnatal and adult life. OPCs express NG2 and platelet-derived growth factor receptor alpha subunit (PDGFRα), two functionally important cell surface proteins, which are also widely used as markers for OPCs. The proliferation of OPCs, their terminal differentiation into OLs, survival of new OLs, and myelin synthesis are orchestrated by signals in the local microenvironment. We discuss advances in our mechanistic understanding of paracrine effects, including those mediated through PDGFRα and neuronal activity-dependent signals such as those mediated through AMPA receptors in OL survival and myelination. Finally, we review recent studies supporting the role of new OL production and “adaptive myelination” in specific behaviours and cognitive processes contributing to learning and long-term memory formation. Our article is not intended to be comprehensive but reflects the authors’ past and present interests.

## 1 Development of the oligodendrocyte lineage

**Fig. 1:**
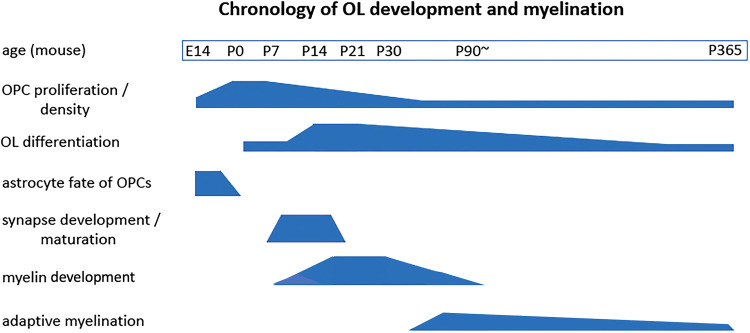
Diagram showing the chronology of OPC proliferation and density, OL differentiation and fate plasticity, synapse formation, myelination, and adaptive myelination. Age of the mouse is indicated across the top. Not drawn to scale.

Myelinating cells arise from committed oligodendrocyte (OL) precursor cells (OPCs) that appear in discrete regions of the ventral ventricular zones (VZ) of the brain and spinal cord during mid- to late gestation, and subsequently in the dorsal VZ (reviewed in references [1], [2]). OPCs reach their peak density by the end of the first postnatal week, and their proliferative activity slowly declines and stabilizes thereafter (Fig. 1). Starting at late embryonic stages, they begin to differentiate asynchronously into OLs, mostly in a caudal to rostral sequence. During the postnatal period of rapid OL production that precedes and accompanies myelination, many of the recently divided OPCs give rise to two differentiated OLs, whereas in the mature central nervous system (CNS) most OPC divisions are self-renewing, generating either one OL and a replacement OPC, or two OPCs [3], [4]. This leads to an age-dependent decline in the rate of OL production (Fig. 1). However, OPCs continue to produce myelinating OLs in the mature CNS; how this is influenced by neuronal activity is under intense investigation (see below, and the article by Karadottir and Monje in this Special Issue). The remainder of this article refers mainly to OL development in mice and a few studies in chick and zebrafish. We believe that much of what we learn from these model species will be generally applicable to all vertebrates, including humans. There certainly are exceptions and some known species-specific differences, which can be informative in their own right, are discussed below.

### 1.1 OPCs originate from multiple germinal zones

There are regional differences in the behaviour of OPCs. For example, both their proliferation and differentiation into OLs occur at a greater rate in white matter than in gray matter [3], [4], [5], [6], [7], [8]. Furthermore, OPCs in the gray matter of prenatal ventral forebrain and spinal cord generate protoplasmic astrocytes in addition to OLs (Fig. 1), whereas those in the dorsal forebrain are restricted to the oligodendrocyte lineage, and OPCs in white matter do not generate astrocytes [3], [9]. These observations have prompted the question of whether OPCs from different germinal zones exhibit different properties.

OPCs from the ventral and dorsal VZ become intermingled in most CNS regions and are indistinguishable in their transcriptomic profiles [10] and ability to generate myelinating OLs during development [11], [12], [13]. However, subtle differences have been reported. For example, dorsally derived OPCs preferentially populate and myelinate dorsal axon tracts [11], [12] and appear to contribute more to remyelination than do their ventrally derived counterparts [13]. The age or replicative senescence of ventrally derived OPCs could contribute to the observed differences (since they first appear before their dorsally derived counterparts), though this has not been tested [14]. OPCs from ventral forebrain maintain functional connectivity with interneurons that originate from the same embryonic germinal zone [15]. Another recent study showed that dorsally derived OPCs that populate the ventral spinal cord after removal of ventral OPCs display altered morphology and differ in their ability to interact with axotomized motor neurons [16]. These studies are beginning to uncover functional differences among OPCs that arise from different germinal zones, including differences in their developmental potential.

### 1.2 The role of PDGF in the development and maintenance of oligodendrocyte lineage cells

**Fig. 2:**
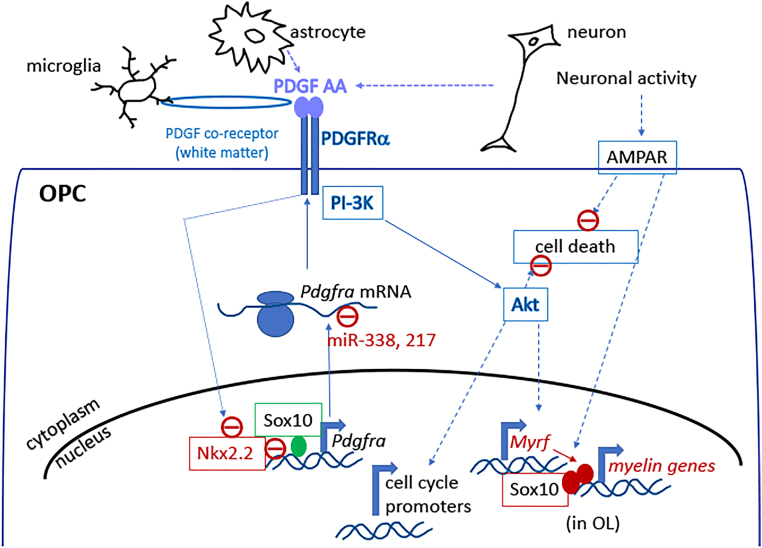
Paracrine and intracellular mechanisms of PDGF signalling in OL lineage cells. PDGF-AA binding to PDGFRα primarily activates Akt in OPCs and promotes their proliferation, survival, and differentiation. How different effector pathways are activated downstream of Akt to achieve these cellular effects remains unknown (dashed lines). For terminal OL differentiation to occur, *Pdgfra* mRNA must be downregulated by Nkx2.2 or miRNAs. Sox10 has a dual role in activating *Pdgfra* transcription in proliferating OPCs (green) and activating myelin gene transcription under the influence of Myrf during OL maturation (red). Outside the cell, neighbouring cells influence the dynamics of OPCs. Microglia in white matter express Nrp1, which acts as a co-receptor for PDGF-AA and potentiates PDGFRα signalling in OPCs [62]. Astrocytes and neurons secrete PDGF, but the specific mechanisms that regulate the secretion or the isoform expressed by them remains unknown. Neuronal activation promotes OL differentiation, possibly through AMPA receptors (AMPAR) (see also Fig. 4), but the molecular signalling pathways downstream of activated AMPARs remain unknown. Arrows indicate the direction of signal transduction and cellular processes. Solid arrows: known effects; dashed arrows: suggested pathways but not experimentally shown; red: factors that are important for terminal OL differentiation; oval: transcriptional factors (green: implicated in OPC proliferation; red: implicated in OL differentiation); red circled dash: inhibition; thick arrows in the nucleus: transcriptional activation.

Commitment to the OL lineage in the mammalian central nervous system is marked by induction of the high mobility group transcription factor Sox10 by the basic helix-loop-helix transcription factors Olig1 and/or Olig2; this occurs immediately prior to the emigration of OPCs from the VZ [17], [18]. Sox10 induces *Cspg4* (encoding the NG2 proteoglycan) and *Pdgfra* [19], [20] (Fig. 2). The subsequent expansion of OPCs and their dissemination through the central nervous system parenchyma is critically dependent on PDGF signalling [21], [22], [23]. However, in some brain regions such as the cerebral cortex and hindbrain, a subset of PDGFRα-expressing OPCs appears to proliferate in the absence of PDGF A-chain [22]. It is possible that PDGF-BB [24] or PDGF-CC [25], [26] might substitute for PDGF-AA in driving expansion of PDGFRα-expressing OPCs in those regions.

PDGF-AA signalling continues to play an important regulatory role in OPC proliferation and/or survival during adulthood. PDGF-AA is known to be limiting in the developing and adult CNS because supplying extra PDGFRα ligand via a transgene driven by the neuron specific enolase (NSE) or glial fibrillary acidic protein (GFAP) promoters increases the number of OPCs in the spinal cord and/or optic nerve [21], [23], [27], [28], [29]. Furthermore, a recent study demonstrated that deleting *Pdgfra* in adult mice caused apoptotic cell death in the majority of OPCs, confirming that continuous PDGFRα-mediated signalling is critical for survival of adult OPCs [30]. The endogenous source(s) of PDGF that triggers PDGFRα signalling need to be re-evaluated. Although early studies had identified astrocytes and neurons as potential sources of PDGF-AA [29], [31], [32], [33], [34], [35] (Fig. 2), *Pdgfa* mRNA was barely detected in astrocytes in a recent transcriptomic study [36]. Perhaps PDGF-CC, which also binds to PDGFRα with high affinity [37], is an astrocyte-derived OPC mitogen. Neurons from early postnatal brain express a high level of *Pdgfa* mRNA [36] and might be an important source of PDGF-AA during development, though the specific site and mechanism of release from neurons remain unknown [34], [35]. In addition, high levels of *Pdgfa* mRNA are detected in microglia and in newly formed OLs [36]. The latter source of PDGF-AA is particularly intriguing, as the processes of newly-differentiated OLs are frequently found closely apposed to those of OPCs [38], perhaps stimulating local divisions to fill gaps in the OPC network created by recent differentiation events. Given the prominent role of PDGF in the development and maintenance of the OL lineage, as well as its potentially important role in remyelination [39], the relative contributions of the different isoforms and sources of PDGF deserve further investigation.

There has been controversy over whether all OLs descend from PDGFRα-expressing OPCs, as described above. A separate population of PDGFRα-negative OL lineage cells, characterized by expression of the DM20 isoform of PLP, has been described in the early developing CNS of chicks and mice [40], [41], [42]. The DM20-expressing cells are prominent in the developing spinal cord, hindbrain and midbrain close to the midline [40], [41], [42]. They do not appear to incorporate BrdU [22], hence might represent early-differentiating, pre-myelinating OLs rather than OPCs [43]. A more recent study showed that in *Pdgfra* conditional knockout (cKO) mice, MBP-expressing OLs appear along the midline of the hindbrain in similar numbers to the DM20-expressing OLs, providing further support for the idea that the caudally located DM20+ OLs arise independently of PDGFRα [44]. In addition, MBP+ OLs appear more rostrally in the corpus callosum and striatum of *Pdgfra* conditional knockout mice by the end of the first postnatal week, initially at a similar density to wild type mice. They do not increase in number beyond P14, whereas OLs in wild type mice undergo a substantial increase in number, presumably due to PDGF-induced proliferation and differentiation of PDGFRα+ OPCs. A small number of myelinated axons are also seen in P16 corpus callosum in an independently generated line of *Pdgfra* cKO mice [45]. These findings suggest the existence of a minor but significant subpopulation of OLs in the forebrain that arises independently of PDGFRα. These might be counterparts of the early-forming DM20+ OL lineage cells that were described in more caudal regions [40], [41], [42]. At present, it is not known whether there are functional differences between PDGFRα-dependent and independent OLs.

Curiously, OPCs in embryonic zebrafish spinal cord do not express PDGFRα [46], [174], so in that sense might resemble the PDGFRα-independent, DM20-expressing OPCs described above. A recent single-cell RNA-sequencing data also revealed little or no expression of *pdgfra* transcript in zebrafish OL lineage cells, whereas *cspg4* was present in specific subclasses of OPCs, mostly those in neuron-rich regions compared to “white matter” areas rich in axons and dendrites [47]. Fish OPCs very likely express DM20, because *Dm20* transcripts appear in small numbers of scattered cells in the ventral hindbrain of zebrafish larvae close to the midline as early as 2 days post-fertilization [48]. Perhaps the PDGFRα-negative DM20-expressing OL lineage of mice [40], [42], [44] is “fish-like”, in that it descends from an early, pioneering OL lineage that first emerged in fish, later to be joined by the tetrapod PDGFRα-expressing lineage that, through its ability to proliferate and migrate extensively, enabled the evolution of larger, more complex brains. This speculative sequence fits with the observation that PDGFRα-independent OLs develop before their PDGFRα-dependent counterparts in mice [40], [42]. Recently, it was shown that an additional developmental adaptation arose later in evolution that increases OL production even more in the human brain [49].

### 1.3 Regional differences in PDGF-AA-dependent OPC proliferation and OL differentiation

After OPCs have populated the CNS by the end of the first postnatal week, PDGF-AA signalling through PDGFRα continues to influence their proliferative behaviour in a region-specific manner [50]. Slice culture studies revealed that OPCs in white matter proliferate in response to exogenous PDGF-AA, whereas those in the gray matter were unresponsive despite similar levels of PDGFRα on their surface [50]. The signals underlying this difference exist locally in the pericellular environment, since OPCs in 300-μm^3^ explants from gray or white matter responded to PDGF-AA similarly to OPCs at the site of origin when heterotopically transplanted into slices or cultured in isolation [50]. Since OPCs are intimately associated with astrocytes, microglia and axons in white matter [51], [52], [53], their enhanced response to PDGF-AA could be imparted by paracrine or contact-mediated effects of their neighbouring cells (Fig. 1). Several extracellular matrix and cell surface proteins affect the function of PDGFRα in different cell types. For example, interaction of PDGFRα with integrins and the extracellular protein tenascin-C modulates the proliferative response of OPCs to PDGF-AA [54], [55]. On endothelial cells, fibroblast growth factor receptor 1 (FGFR1) and PDGFRα heterodimerize in the presence of FGF2, and this could potentiate PDGFRα signalling [56]. On vascular smooth muscle cells, low-density lipoprotein receptor-related protein 1 (LRP1) negatively regulates PDGFRβ signalling, though the effect has not been shown for PDGFRα [57]. Both FGFR1 and LRP1 are expressed on OPCs [36], [58], [59] and could function to locally modulate OPC proliferation [60]. Another cell surface protein neuropilin-1 (Nrp1), which is a co-receptor for vascular endothelial growth factor, interacts with PDGFRα on vascular smooth muscle cells and facilitates PDGFRα activation [61]. In glioma, Nrp1 is expressed by tumour-associated microglia and affects tumour progression [63]. Nrp1 is also expressed by activated microglia and macrophages in the developing and demyelinated corpus callosum brain but not in cortex and facilitates PDGF-AA-mediated OPC proliferation by promoting PDGFRα phosphorylation in OPCs [62], suggesting that trans-activation of PDGFRα on OPCs by Nrp1 expressed by activated microglia contributes to local regulation of OPC proliferation (Fig. 2).

The rate of OPC differentiation into OLs also correlates with the proliferative rate of OPCs and is greater in white matter than in gray matter [3], [4], [5], [6], [7], [8]. Down-regulation of PDGFRα-mediated signalling in OPCs is thought to trigger their differentiation into OLs in vivo (Fig. 2). Timely repression of PDGFRα expression might be mediated by binding of the homeodomain transcription factor Nkx2.2 functioning as a transcriptional repressor at the *Pdgfra* promoter [64], [65] (Fig. 2), and Nkx2.2 expression has been shown to rise in OPCs immediately prior to their terminal differentiation [66], [67]. Additionally, micro-RNA species miR-219 and miR-338 destabilize *Pdgfra* mRNA by targeting the 3’ end of the transcript [68], [69], [70]. Downregulation of *Pdgfra* could partially explain the dichotomy of the effects of signalling through the PI3K-Akt, which promotes proliferation of OPCs and is critical for OL differentiation and myelination [50], [71], [72], [73], [74], [75], [76]. However, the upstream signals that trigger these intracellular signalling events leading to terminal differentiation remain to be clarified (Fig. 2).

### 1.4 Survival of newly generated oligodendrocytes

The time it takes for newly differentiated OLs to mature into myelin-forming OLs varies from one region of the CNS to another. Differentiation appears to be more protracted in the gray matter compared to the white matter, correlating with the rate of OPC division [3], [8]. The period between the final division of an OPC until the newly differentiated OL becomes stably integrated and engages in myelinating axons is a vulnerable period during which the newly formed OL is sensitive to positive and negative influences of their local microenvironment. An early study in the developing optic nerve estimated that ~50% of newly generated OLs undergo programmed cell death, peaking shortly after the first appearance of OLs between postnatal days 3–7 and mainly occurring 2–3 days after the OPC’s final division [77]. It was suggested that over-production and subsequent culling of excess new OLs served to match the OL population to the axons requiring to be myelinated, much as the final number of spinal motor neurons is matched to the muscles that they innervate [78], [79]. Even when OPC production was hugely increased through constitutively elevated PDGF-AA production [21], [23], death of newly-forming OLs increased and the final number of mature myelinating OLs was unaltered. This implies that there is a strong homeostatic mechanism — separate from the mechanisms that control OPC proliferation and survival — that matches myelinating OLs to the requirements of the neural circuitry.

Using EdU pulse labelling combined with genetic fate mapping, we showed that > 80% of OLs in the early postnatal mouse brain are generated within 3–4 days after an OPC division in both cortex and corpus callosum [80]. During this critical temporal window, loss of sensory input caused a specific reduction of newly generated OLs due to increased cell death in the corresponding somatosensory cortex, but not in the motor cortex or the contralateral somatosensory cortex [80]. A similar vulnerability of pre-myelinating OLs expressing the DM20 variant of PLP occurs in the developing rat cortex, where 18–40% of newly-forming DM20+ cells had the appearance of undergoing cell death during the first postnatal month [81]. Long-term live imaging of the neocortex in middle-aged mice revealed that only 22% of newly formed, pre-myelinating OLs become stably integrated and persist as myelin-forming OLs, suggesting that the remaining ~80% of pre-myelinating OLs undergo cell death [82]. Furthermore, this study showed that enhanced sensory input through whisker stimulation can lead to a 5-fold increase in the number of OLs integrated in the whisker field, presumably by enhancing the survival of pre-myelinating OLs, although it is also possible that the local rate of OPC differentiation might increase following whisker stimulation. These observations indicate that OL lineage cells are most sensitive to environmental signals, including and perhaps especially neuronal activity, during the critical time window immediately following the final OPC mitosis and that this period is critical for fine-tuning the number of new OLs that become integrated into developing neural circuitry [83]. Intriguingly AMPA receptors on OL lineage cells appear to be important for the survival of the pre-myelinating OLs (see Section 2.2 below).

Among the genes that are highly expressed during the critical transition from OPCs to early pre-myelinating OLs is transcription factor EB (Tfeb). Tfeb was originally described as a regulator of lysosomal biogenesis and autophagy [84]. However, in the OL lineage it negatively regulates OL survival and myelin formation by transcriptionally activating the pro-apoptotic gene Puma (p53-upregulated modulator of apoptosis), also known as Bcl2-binding component 3 (Bbc3) [85]. This appears to function differently from other inhibitors of myelination, such as Notch activation [86], which acts via DNA-binding protein inhibitors ID-2/4 and Hairy/enhancer of split homologues Hes1/5. Tfeb itself is normally induced by members of the Rag family of GTPases; in zebrafish, *rraga* null mutants are severely hypomyelinated. The myelination defect is partially rescued in *rraga*^*-/-*^*: tfeb*^*-/-*^ double mutants, and *tfeb*^*-/-*^ single mutants make excess myelin [87]. This points to a critical role for Tfeb in regulating apoptotic death and selective elimination of newly-formed pre-myelinating OLs. In future it will be interesting to explore whether and how signals in the microenvironment – including axonal activity-dependent signals – might regulate Tfeb during the critical OPC-to-OL transition.

### 1.5 Age-dependent changes in oligodendrocyte lineage cells

OPCs appear during mid-embryonic stages and persist throughout life. While they maintain the expression of NG2 and PDGFRα and continue to generate myelinating OLs throughout life, their properties gradually change with age. Early studies of OPCs in culture showed that OPCs from perinatal rat optic nerves divide more slowly than those from adult optic nerves [88] and in vivo studies have since confirmed this [3], [4], [5], [6], [8], [89]. Despite this general trend, OPCs are capable of undergoing bursts of rapid proliferation after 6 months of age, resulting in clonal expansion [90]. However, the ability of OPCs to generate OLs that then produce myelin sheaths drops dramatically beyond 12 months of age, resulting in loss of myelin, with impairment in spatial memory [91], [92].

The population of NG2+PDGFRα+ OPCs exhibits a gradient of maturity, based on the expressed genes. For example, the SRY-related HMG box transcription factor Sox2, which is important for self-renewal in embryonic and neural stem cells, is expressed in “immature OPCs” shortly after they are generated from neural progenitor cells in the subventricular zone but is lost with “maturation” of OPCs as they develop and reside progressively more dorsal regions of the corpus callosum [93], [94]. NG2 and PDGFRα expression also declines in OPCs with the age of the animal [95]. Genome-wide transcriptomic analyses also revealed differences in transcripts expressed in OPCs from young and old brains; notably, OPCs from older brains express higher levels of transcripts encoding mature OL and myelin genes than those from early postnatal brains [10], [96]. Furthermore, electrophysiological analysis of OPCs at different ages suggest that their intrinsic membrane properties change dynamically with the state of the cell [97]. Thus, among the OPCs typically identified by the expression of NG2 and PDGFRα there is a range of functional states, from highly proliferative in the developing brain to quiescent in the brains of older mice, and more OL-like transcriptional profile in the more mature brains.

The fate plasticity of OPCs also declines with age. While the majority of OPCs have become largely committed to the OL lineage by birth, a subpopulation of OPCs in the gray matter of embryonic ventral telencephalon and ventral spinal cord generates protoplasmic astrocytes, which co-locate with their OL progeny [3], [9], [98], [99] (Fig. 1). The efficiency of the OPC-to-astrocyte fate switch decreases with postnatal age of the mice. Constitutive *Olig2* deletion in OPCs using *NG2-Cre*, presumably starting around E12 when NG2 begins to be expressed in OPCs that would later colonize the neocortex, results in their conversion to protoplasmic astrocytes by postnatal day 4 (P4), indicating that the fate switch occurs within a week after Olig2 deletion [99]. By contrast, when *Olig2* is deleted in OPCs after weaning, using tamoxifen-inducible *NG2-CreER*, fewer OPCs become astrocytes – and they do so more slowly, over a period of 90 days [100]. The subpopulation of OPCs that differentiates into astrocytes could overlap with the recently identified “pri-OPCs” [101] or “pre-OPCs” that still express Sox2 [93], [94]. The age-dependent decline in OPC fate plasticity appears to be correlated with persistent expression of Sox10 after loss of Olig2 in older mice [100], suggesting a switch from an early Olig2-dependent to an Olig2-independent mechanism of maintaining Sox10 expression as OPCs become irreversibly committed to the OL lineage during the first postnatal month. Epigenetic mechanisms such as histone post-translational modifications in different cell types appear to change dramatically with age [102] and significantly affect OL differentiation [103]. Additionally, ATP-dependent chromatin remodeling factors that interact with Olig2 and/or Sox10, such as Brg1 (Brahma-related gene product 1) of the SFI-SNF family and the chromodomain-helicase-DNA-binding proteins Chd7 and Chd8 [104], [105], [106], [107], [108], could perhaps be integrating intrinsic and environmental age-dependent signals to seal the window of fate plasticity and irreversibly commit to OLs.

## 2 Influence of electrical activity on oligodendrocyte lineage cells

It has been known for many years that OPCs express AMPA-type glutamate receptors, which can influence the proliferation and/or differentiation of OPCs in culture [109], [110]. It was subsequently discovered that OPCs form synapses with axons in the white and grey matter in vivo [50], [111], [112], [113], [114] and receive glutamatergic or GABAergic synaptic input via passing action potentials. This raised the intriguing possibility that OPCs might monitor electrical activity in the axons that they contact and, at some threshold, differentiate and myelinate those active axons in preference to other less-active axons in the vicinity. By speeding conductivity in the newly-myelinated axons, or by promoting axonal energy production or some other beneficial OL-axon interaction, this might be expected to modify and strengthen the firing pattern of circuits and protect them physically and functionally in the long-term, possibly contributing to Hebbian learning and long-term memory in adult animals.

Evidence for activity-related stimulation of myelination has been around for some time – e.g. from experiments in rats that were raised in the dark, which inhibited myelination of their optic nerve axons [115]. There was also evidence that experimental stimulation of cortico-spinal output neurons could augment myelination of descending axons in the spinal cord [116]. Electrical stimulation of mixed neuron-glial cultures subsequently strengthened this evidence [117], [118], as did more recent experiments using optogenetic or chemogenetic approaches to stimulate neuronal activity in live mice [119], [120]. For example, optogenetic stimulation of output neurons in the primary motor cortex enhanced OPC proliferation and myelination of axons in their target areas in the contralateral hemisphere [119]. Drug-induced stimulation of cortical output neurons provided evidence that more and thicker myelin was formed around the drug-activated axonal projections in the corpus callosum and, to a lesser extent, on the axons of adjacent non-activated axons [120] – suggesting that active axons emit short-range diffusible signals to stimulate myelination. Such signals could act on OPCs to induce them to proliferate and differentiate into OLs, or on newly differentiating OLs to enhance their long-term survival and ability to myelinate, or on pre-existing mature OLs to induce synthesis of more internodes, longer internodes or thicker myelin sheaths (more wraps).

### 2.1 Glutamate signalling to oligodendrocyte lineage cells

What are the activity-related myelin-promoting signals? Glutamate is a prime suspect because OPCs and/or OLs express all of the well-known ionotropic glutamate receptor families – AMPA-type receptors (AMPAR), kainate receptors (KAR) and NMDA receptors (NMDAR) – in addition to metabotropic receptors (mGluR). AMPAR are expressed at axon-OPC synapses and are responsible for most glutamatergic (excitatory) synaptic input to OPCs [111], [121]. NMDAR are thought to be expressed in myelinating OLs, where they are thought to monitor neuronal activity by detecting glutamate released from axons into the periaxonal space. The OLs then reciprocate by transferring substrates for energy production into the axons to support their activity [122], [123], [124], [125] (but see reference [126]). KAR and mGluR are presumed to be mainly or exclusively extra-synaptic but little is known about their functions in the OL lineage. The functional outcomes of glutamate signalling to OPCs and OLs are likely to be diverse and are difficult to study because of the multiplicity of receptors and the experimental challenge of separating their activities.

**Fig. 3:**
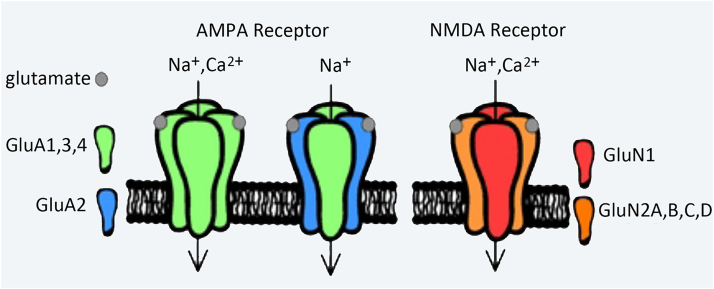
Subunit composition of AMPAR, compared to NMDAR. There are four AMPAR subunits GluA1–4, encoded by *Gria1–4* respectively. Individual subunit form homodimers, and these associate in any combination to form tetrameric receptors composed of one or two subunit species, for example (GluA4_2_ GluA4_2_) or (GluA2_2_ GluA4_2_). OL lineage cells express GluA2, GluA3 and GluA4, but not GluA1. Following binding of glutamate from neurons, the AMPAR channel opens and triggers membrane depolarization through influx of Na^+^ and Ca^2+^ ions – unless GluA2 is present, when Ca^2+^ entry is blocked. Genetic experiments in mice indicate that AMPAR activation stimulates survival of pre- or newly-myelinating OLs, regardless of whether AMPAR contains GluA2 [121] (however, see reference [138]). Also illustrated is NMDAR, which is also a “dimer of homodimers”, of which GluN1 is an obligatory subunit. OL lineage cells also express NMDAR, activation of which is thought to control glucose uptake and the support of axonal metabolism by myelinating OLs [124]. However, conditional deletion of the obligatory subunit GluN1 from the OL lineage was reported to be phenotypically neutral [126].

A number of in vitro and in vivo studies over the years have concluded that glutamate signalling influences OL development. However, there has been a striking lack of consensus as to whether the primary effect is on OPC proliferation or migration, OL differentiation or survival or something else [110], [127], [128], [129]. By their nature these studies could not distinguish direct from indirect effects (e.g. through glutamate-mediated effects on neighbouring cells such as astrocytes), nor could they define the specific receptors involved. With the hope of introducing some clarity we embarked on a series of genetic experiments to investigate the role of glutamate-mediated synaptic signalling in postnatal OL development and myelination; to avoid ambiguity, we focused on AMPAR-mediated synaptic signalling by knocking out AMPAR subunits individually and in combination in mice [121] (Fig. 3).

### 2.2 AMPA receptor-mediated regulation of oligodendrocyte development

AMPAR is a homo- or hetero-tetramer constructed as a “dimer of homodimers” of any combination of the four subunits GluA1–4 (e.g. A4_4_ or A2_2_-A4_2_) (Fig. 3)*,* encoded in the genome by *Gria1–4* respectively*.* Expression studies of OL lineage cells in vitro [109] and in situ [121] imply that OPCs and/or newly-differentiating OLs express *Gria2–4* but not *Gria1*. It was known that germline *Gria3-*null mutants display little overt physiological or behavioural phenotype [130]. We also could find no electrophysiological or developmental phenotype among OL lineage cells in the white matter of *Gria3-*nulls in situ at P14: no reduction in kainate-induced current compared to wild type, no deficits in the number or proliferation rate (EdU labeling index) of OPCs, number of differentiated OLs, number of myelin sheaths or myelin thickness (g-ratio) [121]. Moreover, deleting *Gria2* conditionally on its own (with *Sox10-Cre*) also had no effect on OL development, according to the criteria listed above. This was unexpected, because AMPARs that contain GluA2 (Q583->R edited isoform) have distinct electrical properties, being impermeable to Ca^2+^ ions. Thus, our experiments imply that OL development is insensitive to the Ca^2+^ permeability properties of AMPAR.

OL lineage-specific knockout of *Gria2* on the *Gria3-*null background (*Gria2/3* double-KOs) resulted in ~70% reduction in the frequency of miniature excitatory post-synaptic currents (ePSCs) induced in patch-clamped OPCs in the subcortical white matter by bath application of Ruthenium Red (RR). There was no change in the amplitude of individual ePSCs, suggesting that fewer AMPAR-containing axon-OPC synapses were formed or maintained in *Gria2/Gria3* double-mutants than in *Gria3-*null controls. *Gria2/3* double-KOs had normal numbers of OPCs and normal rates of OPC proliferation, but ~25% less differentiated CC1^+^ OLs and a similar reduction in myelin sheaths in the early postnatal (P14) corpus callosum. This resulted from increased apoptotic death of newly forming pre-myelinating OLs, judged by co-labelling for activated Caspase 3 and *Enpp6* [121], [131]. However, the OL deficit resolved in the following weeks and by P70 the numbers of OLs and myelin sheaths in the double mutant were indistinguishable from controls.

*Gria2/3/4* triple-KO OPCs had almost no RR-evoked ePSCs (~1% of control), implying near-complete elimination of AMPAR-containing synapses. The OL phenotype of triple-KOs was similar to that of *Gria2/3* double-KOs, in that ~22% less CC1^+^ OLs accumulated in the subcortical white matter in the early postnatal period. However, in triple-KOs the deficit of differentiated OLs and myelin persisted in the longer term; we measured ~26% reduction in OLs at P53 [121] and ~15% reduction in both OLs and myelin sheaths at P93 (T.S. and W.D.R. unpublished). In neither the double-KO nor the triple-KO was there any change in the number or length of internodes made by individual OLs, nor an altered g-ratio. Thus, any activity-dependence of these morphological features of OLs and myelin [119], [120], [132], [133], [134], [135] must be mediated by glutamate receptors other than AMPAR, or by signals other than glutamate.

In summary, we showed that AMPAR-mediated synaptic signalling to OPCs and/or pre-myelinating OLs regulates myelination of the callosal white matter by stimulating long-term survival and integration of newly-differentiating OLs, without influencing myelin synthesis by individual OLs. We were unable to detect any effect on OL numbers in the cortical gray matter of *Gria2/3* or *Gria2/3/4* mutants [173], presumably because most myelinating OLs in the cortex are associated with GABAergic inhibitory interneurons, not glutamatergic neurons [136], [137].

Our conclusion that Ca^2+^ permeability is not important for the developmental effects of AMPAR activation is seemingly at odds with a study in which mutant GFP-tagged GluA2 subunits were targeted to OPCs in the postnatal corpus callosum by stereotaxic injection of retrovirus vectors [138]. (Retroviruses infect and replicate in cells that are actively dividing, hence the preference for OPCs.) One such retrovirus encoded GluA2(Q583), a constitutive “unedited” form of GluA2 designed to cause AMPARs in which it incorporates to be Ca^2+^-permeable. This perturbation increased the proportion of infected OPCs that incorporated EdU and reduced the proportion of EdU^+^ CC1^+^ newly-formed OLs, relative to those infected with a control GFP-expressing retrovirus, leading the authors to propose a key role for GluA2(R583) in stimulating OPC division and shifting the balance between proliferation and differentiation. There are many reasons why this gain-of-function approach and our own loss-of-function experiments might lead to divergent conclusions. As pointed out by Chen et al. [138], it is conceivable that early loss of one or more GluA subunits in our experiments might lead to compensatory up-regulation of other signalling pathways, obscuring an important role of AMPAR in controlling OPC proliferation (note, however, that *Gria1* was not upregulated in the absence of GluA2/3 [121]). On the other hand, dominant gain-of-function approaches qualitatively alter the signalling system they are intended to probe, which can lead to unanticipated side-effects that undermine interpretation.

**Fig. 4:**
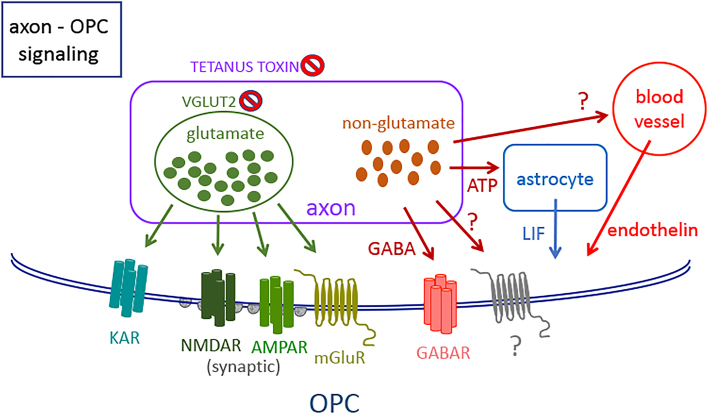
Activity-dependent axon-OPC signalling. Axons release neurotransmitters such as glutamate, GABA or ATP, depending on neuronal subtype, whenever they fire an action potential. Glutamate released from axons in white matter or gray matter can act directly on synaptic (AMPAR or NMDAR) or extra-synaptic (mGluR or KAR) glutamate receptors on OPCs. ATP can act indirectly on OPCs via astrocytes, which release leukemia inhibitory factor (LIF) to stimulate myelin synthesis [118]. Unknown factors from active axons stimulate release of Endothelin from cells in blood vessels, which influences the number of myelin internodes synthesized by individual OLs [135]. There are undoubtedly other activity-dependent signals that act either directly or indirectly on OPCs. Tetanus toxin expression in neurons blocks all synaptic and non-synaptic vesicular release [134], and genetic ablation of vGlut2 specifically blocks glutamate release by preventing its loading into synaptic vesicles [129]. Pharmacogenetic approaches cannot yet target specific signalling pathways or receptor subtypes; this requires systematic targeting of individual receptors, e.g. by conditional gene deletion in OL lineage cells. Using the latter approach, we showed that AMPAR-mediated signalling stimulates myelin production during postnatal development by enhancing the survival of newly-forming OLs [121].

The data summarized above imply a subtle regulatory role for AMPAR in developmental myelination – not an all-or-none effect. This is not surprising, because there are many other activity-dependent signalling pathways that can also contribute. Apart from the alternative glutamate receptors such as NMDAR, KAR and mGluR, which are also expressed in OL lineage cells, there are other myelin-promoting signals such as leukemia inhibitory factor (LIF) and Endothelin, release of which (from astrocytes and endothelial cells, respectively) can also be stimulated by neuronal activity [118], [135] (Fig. 4). Nevertheless, the seemingly modest scale of the effects of GluA deletion on developmental myelination might underplay the significance of AMPAR signalling in adulthood. It is thought that there might be two modes of myelination — 1) constitutive and 2) activity-dependent [139], [140], [141], [142] — and that Neuregulin and/or Brain-derived neurotrophic factor (BDNF) can switch between them [139]. BDNF, acting through the TrkB receptor on OPCs, has also been implicated in activity-dependent myelination and repair during adulthood [143], [144]. It could be that the constitutive mode of myelin production predominates during early postnatal development, because there must be strong selective pressure for rapid myelination of circuits that are essential for basic life processes (e.g. sibling competition and survival in the nest). Only after that might activity-dependent myelination come into its own, as circuits are selected and refined for learned sensorimotor behaviours such as detection and escape from predators, reproduction, foraging for food and other essential adult skills. If so, the relative importance of AMPAR-dependent myelin development might increase in adulthood.

A recent live-imaging study, in which OL lineage cells and myelin were followed over weeks and months by two-photon microscopy, showed that housing mice in an enriched environment dramatically increased the number of newly-differentiating OLs that survived and formed myelin on the axons of glutamatergic projection neurons in the upper layers of the somatosensory cortex [82]. Remodeling of pre-existing OLs or myelin internodes was observed infrequently in those experiments, so the majority of new myelin that formed in response to environmental enrichment (in mice aged 2 months to 2 years) was a result of enhanced long-term survival and integration of newly generated OLs. This finding, together with our genetic studies of AMPAR discussed above, suggests that experience-dependent OL plasticity might be driven mainly by AMPAR-mediated synaptic signalling. This can be tested in future, using inducible Cre driver lines such as *Pdgfra-CreER* to drive tamoxifen-dependent knockout of GluA subunits in adult OPCs, in order to examine their roles in adult OL genesis and OL-dependent behaviours (see next section).

## 3 Adaptive myelination, learning and memory

It is now clear that artificially manipulating axonal activity or activity-dependent signalling can modify patterns of myelination in vivo [116], [119], [120], [133], [134], [140], [145], [146], [147]. This activity-dependent OL plasticity is presumed to be beneficial for the animal and has been labelled “adaptive myelination”, implying that it can modify the sensory input and/or motor output circuitry to provide a survival advantage (reviewed by [148], [149], [150]). We and others have tested this idea through behavioural experiments in mice. We chose to focus on motor skill learning in the first instance [131], [151].

### 3.1 New oligodendrocyte generation is required for motor learning and memory

Motor skill learning is a form of unconscious “non-declarative” or “intrinsic” learning, in that it gives rise to a long-term memory that can only be demonstrated through replay of the learned skill. By contrast, “declarative” or “explicit” learning involves e.g. new concepts or abilities that can be described verbally. Non-declarative learning is an ancient system that includes associative learning in invertebrates (e.g. the gill retraction reflex of marine molluscs) and Pavlovian conditioning. It is known to reflect changes to the intrinsic properties of the circuits that drive the behaviour, not something that is formed or stored remotely [152]. The generally accepted mechanism of intrinsic learning in both invertebrate and vertebrate animals involves synaptic strengthening. In vertebrates, however, it is perfectly conceivable that it could also reflect adaptive alterations to myelin.

That myelination might play a role in intrinsic learning was first suggested by the discovery that acquiring a new motor skill, such as playing the piano or juggling, was accompanied by micro-structural changes in white matter tracts, detected by magnetic resonance imaging (MRI) [153], [154]. Moreover, in rats that mastered a skilled one-handed reaching/grasping task, an altered MRI signal in the contralateral corpus callosum was accompanied by increased Myelin basic protein (Mbp) immunoreactivity, suggesting that motor learning is accompanied by elevated myelin synthesis [155].

To test the idea that de novo myelination is *required* for motor learning we inhibited the generation of new OLs in adult (6–9 week-old) mice, by tamoxifen-dependent knockout in OPCs (using *Pdgfra-CreER*) of the transcription factor Myrf, which is required to orchestrate the myelination program in newly-differentiating OLs. This caused OLs that were newly-differentiating from OPCs to stall at a pre-myelinating stage, undergo apoptosis and be cleared by microglia and/or other cells. The resultant failure to generate new OLs prevented *Myrf-*cKO mice from learning to run at speed on a “complex running wheel” with unevenly spaced rungs [151]. If the cKO mice learned to run on the complex wheel before tamoxifen administration, then they retained their ability to run on the wheel post-tamoxifen. This made two important points: 1) ongoing OL production is not required to recall and perform a pre-learned skill, only to learn the skill in the first place, and 2) loss of Myrf does not compromise the animals’ physical ability to run on the wheel, e.g. through diminished cardiovascular or muscular tone. Learning to master the complex wheel stimulated OL generation in the corpus callosum and overlying motor cortex, but not in the optic nerve or visual cortex, demonstrating regional specificity. OL generation was stimulated in the corpus callosum only the first time the mice encountered the wheel, not when re-exposed to it a second time after a break of 3 weeks, supporting the idea that novel experience (learning) was the stimulus for increased OL generation, not physical activity per se.

We subsequently showed that the running performance of *Myrf*-cKO mice on the complex wheel fell below that of control littermates surprisingly quickly – within just a few hours of starting to self-train on the wheel [131]. This diminished ability to learn was accompanied, over a similar time-scale, by a reduced rate of OL production – judged by in situ hybridization for *Enpp6*, a transiently-expressed marker of newly-differentiating OLs [131]. This cemented and extended the conclusions of McKenzie et al. [151] that 1) learning a new motor skill stimulates OL development rapidly in task-relevant brain regions and 2) the additional OLs so generated are necessary for motor learning and formation of long-lasting motor memory.

### 3.2 Fear conditioning, spatial learning and long-term memory formation

Recently, a similar *Myrf-cKO* model (using *NG2-CreER* rather than *Pdgfra-CreER*) was used to investigate the potential role of OL generation in contextual fear conditioning – another example of intrinsic learning – in which mice learn to associate a benign conditioning stimulus (pre-exposure to the experimental cage setup) with a subsequent aversive stimulus (mild electrical foot-shock). After about one week of pre-conditioning, exposure to the benign stimulus elicits fearful behaviour (freezing) in the absence of the foot shock. Wild type mice can retain this associative “fear memory” for more than 30 days; the *Myrf*-*cKO* mice retained the memory for 24 h but could not preserve it long-term [156]. Thus, new OL genesis is required for formation, consolidation or recall of long-term, “remote” fear memory.

The fear-conditioning paradigm increased proliferation of OPCs in the prefrontal cortex (PFC) within 24 h and the additional OPCs gave rise to increased numbers of differentiated OLs and myelinated axons over the following weeks. This differs from our experience with the complex wheel, in that we did not observe increased OPC proliferation until 4 days after first encounter with the wheel – as if it were a secondary response, perhaps to replenish OPCs recently lost by differentiation into OLs [151]. Apart from the obvious difference between our learning paradigms, Pan et al. [156] focused on the grey matter of the prefrontal cortex whereas we focused on callosal white matter. Since a significant fraction of all myelin in cortical grey matter (~50% in layers 2/3) is associated with axons of Parvalbumin (PV)-positive GABA-ergic inhibitory interneurons [136], [137], whereas axons in the corpus callosum are predominantly glutamatergic, axon-OPC interactions in the two regions are likely to be rather different.

Pan et al. [156] looked for functional correlates of the loss of new myelin formation in *Myrf-cKO* mice by examining by c-Fos immunoreactivity in neurons, a marker of neuronal activation. They found a reduction in the density of c-Fos positive neurons in several fear-relevant brain regions (e.g. PFC, amygdala, hippocampus) during recall of remote fear memory, but not during recent (24 h) recall. They also examined Ca^2+^ dynamics optogenetically, using the calcium indicator GCaMP6f delivered to the PFC via an AAV vector. This showed, in confirmation of previous electrophysiological data, that PFC activity was suppressed in wild type mice during recent recall of fear memory compared to mice that did not undergo fear conditioning. However, the PFC circuitry evolved over time post-conditioning so that during remote (30 day) recall, neuronal activity became elevated rather than suppressed. In *Myrf-cKO* mice this long-term reorganization of PFC network activity did not occur, although there was no difference in PFC activity between *Myrf-cKO* and wild type mice either during initial conditioning or recent recall. This starts to link adaptations at the level of OLs and myelin to changes in the properties of neural circuits required for establishment of long-term memory.

Steadman et al. [157] independently used the *Myrf-cKO* model (with *NG2-CreER*) to investigate the role of adult OL genesis in fear conditioning and memory. They too found that new OL production is required for consolidation of remote fear memory (tested at 28 days post-conditioning), but not for initial conditioning or short-term (24 h) memory. Steadman et al. [157] also found that active myelination is required for consolidation or recall of long-term spatial memory in the Morris water maze test but, similar to fear conditioning, not for spatial learning per se, or for short-term recall. They went on to investigate brain oscillatory activities that are widely held to participate in off-line consolidation of recently-acquired memories. Rhythmic firing of sets of neurons gives rise to different patterns of oscillatory extracellular field potential, defined by frequency – e.g. theta waves (4–8 Hz) and gamma waves (25–100 Hz) in the hippocampus and elsewhere. It has been proposed that adaptive myelination, by altering conduction speeds, might serve to modulate the frequency or amplitude of such oscillations and/or the coherence of different oscillators, on which neural computation is thought to depend [148], [158]. Memory consolidation relies mainly on oscillatory firing patterns that occur during periods of immobility, especially non-REM sleep (also known as slow wave sleep or deep sleep). These include large-amplitude “sharp waves” and associated high-frequency bursts (“ripples”, 140–220 Hz) in hippocampal CA1, and “spindles” (12–14 Hz trains of short duration) in thalamo-cortical circuits. The hippocampal sharp wave/ripple (SWR) events and cortical spindles interact, in that the timing of these events becomes more closely correlated during memory consolidation [159]. Strikingly, Steadman et al. [157] found that *Myrf-cKO* mice did not undergo this SPW-R/spindle temporal entrainment following fear conditioning, implying that adaptive OL genesis is key to the regulation of ripple-spindle coupling.

How could OL/myelin production influence this coordinated activity across the brain? One obvious idea is that myelination synchronizes transmission times of axonal connections between hippocampus and cortex, allowing local circuits in those regions to fire in concert. Consistent with this idea, greater variability in transmission times of long-range thalamo-cortical connections was observed in mice with subtly impaired myelin [160]. Alternatively, both hippocampal SWR and cortical spindle events could have a common driver, e.g. they might be controlled separately by third-party neurons, and myelination might fine-tune transmission times in the separate arms of the circuit. Long-range innervation from cholinergic neurons in the forebrain medial septum is believed to drive hippocampal theta waves, for example [158]. A third possibility is that myelination of inhibitory PV interneurons might fine-tune negative feedback loops that are responsible for local control of oscillatory behaviour [161]. These ideas are neither mutually exclusive nor exhaustive.

### 3.3 Working memory training

We have been interested to discover whether adaptive OL genesis and myelination is more broadly involved in other “cognitive” forms of learning and memory. That it might be, is suggested by MRI studies that reveal alterations of white matter microstructure as people learn a second language [162] or undergo working memory training [163]. Working memory is a limited capacity storage system that is used to hold and manipulate information over short periods of time [164], [165]. Working memory capacity can be increased through training, and correlates closely with measures of “general intelligence” in humans [166]. We trained mice in a T-maze “rewarded alternation” task that relies mainly on short-term memory function, arguably analogous to human working memory. In this task, mice must remember which of two maze arms they visited to receive a food reward in an initial trial, in order to correctly select the opposite arm – and receive a second reward – in a subsequent trial 30 s later. Wild type mice improve their performance in this test over an ~8 day period (ten paired trials per day), but we found that *Myrf-cKO* mice failed to improve noticeably over the same period (T.S. and W.D.R. unpublished). This suggests that the neural circuitry that holds short-term working memory in mice can undergo training-induced myelination, as a result of which it acquires a higher holding capacity and/or span. In keeping with this idea, we found that memory training is accompanied by increased production of newly-differentiated OLs in the anterior corpus callosum at the level of the anterior cingulate cortex, which is known to be active during the exercise of working memory (Shimizu et al., in preparation).

Therefore, there is increasing evidence that OL generation is both stimulated and required for training-induced learning and memory processes. In the motor skill domain, OL genesis is needed both for initial learning and the formation of long-term motor memory [131], [151]. In contextual fear conditioning or spatial learning, new OL genesis is not needed for initial learning but is required to lay down remote fear or spatial memories [156], [157]. Contextual fear conditioning and spatial learning are both hippocampus-dependent learning processes, whereas motor skill learning is independent of the hippocampus. For example, the celebrated Henry Molaison, who had his hippocampus bilaterally resected to relieve severe epilepsy, was still able to master a mirror-writing fine-motor task [152] and reportedly also could learn new dance steps. Perhaps, therefore, new OL generation is especially important for hippocampus-independent learning, as well as formation and maintenance of long-term memories (also see Section 4, Conclusions and Future Directions).

### 3.4 How do new oligodendrocytes contribute to circuit plasticity, learning and memory?

Different properties of OLs might come into play at different stages of the learning process. For example, Xiao et al. [131] found that newly-differentiating OLs are required within hours of mice starting to self-train on the complex wheel for optimal early-stage learning. It is unlikely that this early requirement for OLs reflects compact multi-lamellar myelination per se, but might involve another function such as the first ensheathing wrap of the axon, induction of sodium channel clustering along the axon prior to myelination [167], or metabolic coupling between pre-myelinating OL and axon [122], [123], [124]. The ultimate function of OLs – to form compact myelin – is likely to be important in the later stages of learning and memory formation. It is an attractive idea, for example, that the extreme longevity of myelinating OLs – which, once formed, can survive for the lifetime of the animal [168], [169] – is what underpins the consolidation and preservation of all kinds of lasting memories. This could also include the life-long social conditioning that is normally established during early postnatal life by intra-species contact and has been shown to depend critically on myelination in the prefrontal cortex [170], [171].

It could turn out that the relative importance of “early” and “late” OL functions depends on the learning paradigm under study. This will need to be investigated in future using genetic mouse models other than *Myrf-cKOs*. Loss of Myrf causes an early failure of OL differentiation, leading to death of pre-myelinating OLs and clearance by microglia or other cells, so it is not possible to assess the roles of different stages of OL development and myelination using this approach. We need to develop alternative models that do not result in early OL death and clearance, but rather arrest OL differentiation at specific pre-myelinating stages, or disrupt distinct biological functions of OLs.

## 4 Conclusions and future directions

We have described recent findings on how intrinsic and extrinsic mechanisms regulate OL dynamics at different stages of maturation at different development ages (Fig. 1), with an emphasis on two signalling pathways – PDGF/PDGFRα and glutamate/AMPAR. PDGF is one of the primary mitogens responsible for expanding the OPC population during development, for homeostatic control of OPC number during adulthood and in response to OL death and demyelination. Down-regulation of the PDGF signalling pathway is believed to be critical for timely OL differentiation and myelination. Trans-activation of PDGFRα on OPCs by a neighbouring microglial subpopulation contributes to regional differences in the rates of OPC proliferation and OL differentiation (Fig. 2)**.** Recent genetic studies have provided new evidence for a subset of OLs that appears to arise independently of PDGFRα, possibly corresponding to a previously described population of early-generated DM20+ OLs. We speculate here that the PDGF-independent OLs are “primitive”, in that they evolved and are present in fish species, and that these were joined by PDGF-dependent OLs at the fish-tetrapod transition. Further studies are needed to elucidate whether and how PDGFRα-independent OPCs proliferate and whether PDGFRα-dependent and -independent OPCs generate transcriptionally [172] and functionally distinct OL subpopulations. This could have important implications for the etiology and repair of myelin-related diseases.

After developmental myelination is completed, OPCs persist and continue to generate OLs that myelinate axons, in an activity-dependent manner. We have focussed on AMPAR-signalling as a likely key mediator of activity-dependent OL dynamics (Fig. 3, Fig. 4). The evidence so far indicates that AMPAR-signalling controls survival of pre-myelinating OLs, thereby regulating the generation of new myelin in development. However, the specific intracellular signalling mechanisms that transduce AMPAR signalling are unknown (Fig. 2). It remains to be seen whether glutamate/AMPAR also regulates new myelin production in adults, contributing e.g. to learning and memory. There are undoubtedly many signalling pathways, some already known (e.g. GABA, endothelin, adenosine) and others yet to be identified, that orchestrate different aspects of activity-dependent myelin production or remodelling — e.g. myelin sheath length or thickness or other morphological features of the axon-myelin unit. There is a large amount of work remaining to be done in this area.

Motor skill learning depends on new OL formation. Spatial or fear learning do not, seemingly, although long-term preservation of fear or spatial memories do require OL production and presumably myelination. This points to mechanistic differences among distinct learning modalities. Acquisition of a new motor skill seems to go hand-in-hand with long-term retention of the skill, as if both learning and long-term motor memory formation depend on one and the same OL-dependent process(es). In contrast, spatial or fear learning and their associated long-term memory traces rely on sequential OL-independent and OL-dependent processes, recalling the long-standing observation that spatial learning and short-term memory formation occur in the hippocampus but the spatial memory is transferred elsewhere (e.g. to the cortex) for long-term storage. Future experiments can be designed to explore such ideas and hence increase our understanding of the role of OLs in learning and memory processes and start to relate these to human psychology, physiology and pathophysiology. We also need to probe more deeply into the functional contributions of OL lineage cells at different stages of learning and memory formation, e.g. by devising genetic means of uncoupling different developmental stages or functions of OLs.

## Declaration of Competing Interest

The authors declare that they have no known competing financial interests or personal relationships that could have appeared to influence the work reported in this paper.
